# Impact of Right Heart Failure on Clinical Outcome of Left Ventricular Assist Devices (LVAD) Implantation: Single Center Experience

**DOI:** 10.3390/healthcare10010114

**Published:** 2022-01-06

**Authors:** Dusko Terzic, Svetozar Putnik, Emilija Nestorovic, Vladimir Jovicic, Dejan Lazovic, Nemanja Rancic, Vladimir Milicevic, Dragan Ivanisevic, Radmila Karan, Aleksandar Mikic

**Affiliations:** 1 Department for Heart Transplant, LVAD and ECMO, Clinic for Cardiac Surgery, University Clinical Center of Serbia, 11000 Belgrade, Serbia; svetozar073@yahoo.com (S.P.); emanestor@gmail.com (E.N.); medi@eunet.rs (V.J.); lazovic.dejan88@gmail.com (D.L.); vladodrillwork@gmail.com (V.M.); dusan-ivanisevic@hotmail.com (D.I.); karan.radmila@gmail.com (R.K.); mickation@yahoo.com (A.M.); 2 Medical Faculty University of Belgrade, 11000 Belgrade, Serbia; 3 Faculty of Medicine of the Military Medical Academy, University of Defence, 11000 Belgrade, Serbia; 4 Centre for Clinical Pharmacology, Military Medical Academy, 11000 Belgrade, Serbia

**Keywords:** left ventricular assist devices, end-stage heart failure, right ventricular failure, right heart failure, treatment

## Abstract

The aim of this study was to examine the incidence and significance of right heart failure (RHF) in the early and late phase of left ventricular assist device (LVAD) implantation with the identification of predictive factors for the development of RHF. This was a prospective observational analytical cohort study. The study included 92 patients who underwent LVAD implantation and for whom all necessary clinical data from the follow-up period were available, as well as unambiguous conclusions by the heart team regarding pathologies, adverse events, and complications. Of the total number of patients, 43.5% died. The median overall survival of patients after LVAD implantation was 22 months. In the entire study population, survival rates were 88.04% at one month, 80.43% at six months, 70.65% at one year, and 61.96% at two years. Preoperative RHF was present in 24 patients, 12 of whom died and 12 survived LVAD implantation. Only two survivors developed early RHF (ERHF) and two late RHF (LRHF). The most significant predictors of ERHF development are brain natriuretic peptide (BNP), pre-surgery RHF, FAC < 20%, prior renal insufficiency, and total duration of ICU stay (HR: 1.002, 0.901, 0.858, 23.554, and 1.005, respectively). RHF following LVAD implantation is an unwanted complication with a negative impact on treatment outcome. The increased risk of fatal outcome in patients with ERHF and LRHF after LVAD implantation results in a need to identify patients at risk of RHF, in order to administer the available preventive and therapeutic methods.

## 1. Introduction

Due to the limited number of available donors and the limited effectiveness of conservative treatment methods, left ventricular assist devices (LVADs) are widely used today to treat patients with end-stage heart failure [1]. Good therapeutic results are achieved as a result of the continuous and intensive development of new devices, improvement of surgical techniques, accumulation of experience in implantation, and improved post-implantation clinical management [2,3]. The development of experienced and well-trained teams composed of cardiac surgery and cardiology specialists, LVAD technicians, perfusionists, and nurses reduces the rate of complications following LVAD implantation [4].

The importance of right ventricular failure (RVF) is reflected in the fact that it can complicate up to 40% of LVAD implants and is associated with increased morbidity, mortality, prolonged hospital stays, frequent hospitalizations, and increased treatment costs [5].

The physiological and pathophysiological effects of LVAD implantation on the right ventricular (RV) function have been explained in the literature, significantly contributing to better treatment results [6]. However, the identification of patients at increased risk for post-implantation right heart failure (RHF) is still the subject of intensive research [7].

The aim of this study was to examine the incidence and significance of RHF in the early and late phases of LVAD implantation with the identification of predictive factors for the development of RHF. Furthermore, it evaluated the impact of preoperative RHF on the LVAD implantation outcome. The RHF defining criteria were adopted from the Interagency Registry for Mechanically Assisted Circulatory Support (INTERMACS) definition to accommodate our study follow-up model [8,9].

## 2. Materials and Methods

This was a prospective observational analytical study consistent with a cohort study type. From June 2013 to March 2021, 97 devices for permanent mechanical circulatory support (MCS) were implanted at the Cardiac Surgery Hospital of the Clinical Center of Serbia. Out of these cases, the study included 92 patients who underwent LVAD implantation and for whom all necessary clinical data from the follow-up period were available, as well as unambiguous conclusions by the Heart Team regarding pathologies, adverse events, and complications. All patients (or their family members) signed the informed consent.

The RHF criteria were adapted from the INTERMACS definition, requiring seven days of support and consisting of two criteria: (1) records of elevated central venous pressure (CVP) by direct measurement (CVP or right atrial pressure [RAP] >16 mmHg) or dilated inferior vena cava without any inspiratory variation or elevated jugular venous distension; (2) manifestations of elevated central venous pressure characterized by peripheral edema (>2 either new or unresolved), presence of ascites or palpable hepatomegaly (physical examination or diagnostic imaging), or laboratory evidence of hepatic (total bilirubin > 34 µmol /L) or renal dysfunction (creatinine > 176 µmol /L).

The early RHF and late RHF were defined by meeting these criteria 7–14 days and >14 days after surgery, respectively. Preoperative RHF was defined by the same criteria on the day of surgery [8,9]. When RHF was reported as early RHF and/or persisted more than 14 days after implantation of LVAD, it was categorized as early RHF.

We considered such an adapted definition of the RHF suitable because it provides a conclusion on the clinical condition without categorizing RHF according to the severity of the clinical picture, while the treatment strategy is performed on a case-to-case basis and their actual clinical findings. Furthermore, such a definition of late RHF is suitable for the long-term follow-up considering the secondary objective of this study was to show the incidence of late RHF over a more extended follow-up period.

The presence of RHF was identified based on the criteria mentioned above by a cardiologist and cardiac surgeon and entered into the database before surgery, and at seven days, 14 days, one month, 3, 6, and 12 months, and at each subsequent yearly scheduled and unscheduled follow-up visit. The primary outcome was patient survival after LVAD implantation and the development of RHF in the early and late post-implantation period and their impact on survival. We believe that the relationship between these two outcomes adequately represents the importance of RHF.

All LVAD devices were implanted on a beating heart using an extracorporeal circuit. Before surgery, patients underwent the standard cardiac surgery clinical workup with an emphasis on cardiac function (brain natriuretic peptide [BNP], echocardiography, hemodynamic evaluations), followed by liver and renal function assessment. Neurological status and psychological assessment of the compliance for LVAD implantation and subsequent post-implantation challenges were observed in all study patients.

Left ventricular (LV) dimensions, as well as LV systolic function (LV EF), were measured with a transthoracic two-dimensional echocardiographic examination according to a standard protocol. The LV end-systolic diameter (LV ESD) and end-diastolic diameter (LV EDD) were measured using a two-dimensional parasternal longitudinal section and M-mode echocardiography. The LV EF was calculated using Teicholz and Simpson methods. Right ventricular (RV) dimensions and systolic and longitudinal RV function were assessed qualitatively and quantitatively using a two-dimensional apical four-chamber cross-section, parasternal longitudinal section, and parasternal cross-section. Measurements included the amplitude of tricuspid annulus plane systolic excursion (TAPSE), the maximum tricuspid annulus (TA) systolic velocity, and the RV systolic and diastolic fractional area change (FAC), tricuspid regurgitation (TR), and right ventricular systolic pressure (RVSP) [10]. The parameters that characterized the RV failure and were thus subject to our special attention included: FAC < 20%, TAPSE < 15 mm, tricuspid annulus systolic motion < 10 cm/s, RVSP < 40 mmHg, CVP or RAP > 16 mmHg and RAP/PCWP > 0.63. It may be important to emphasize that echocardiographic monitoring was performed daily during the immediate postoperative course, enabling RV load determination, and helping clinicians adjust the intensity of pump operation and administration of inotropic agents and/or treatment for pulmonary hypertension.

Preoperatively, all patients underwent right cardiac catheterization providing data related to cardiac output (CO [L/min]), cardiac index (CI [L/min/m^2^]), right atrial pressure/central venous pressure (RAP [mmHg]), pulmonary artery wedge pressure (PAWP [mmHg]), mean pulmonary artery pressure (mPAP [mmHg]), pulmonary vascular resistance (PVR [WU]), and transpulmonary gradient (TPG [mmHg]).

Liver function was evaluated using the levels of aspartate aminotransferase (AST), alanine aminotransferase (ALT), and total bilirubin. Renal function was evaluated using the levels of serum creatinine (mmol/L), urea (mmol/L), and glomerular filtration rate (eGFR [mL/min/1.73 m^2^]). During the post-implantation period after discharge, all preoperative parameters except hemodynamic measurements were acquired consistently by the same heart team as before LVAD implantation at the following time points: 3, 6, and 12 months, and at each subsequent yearly scheduled and unscheduled follow-up visit. All data were collected from the archived records of regular follow-up visits. The diagnosis of RHF was made by cardiology and cardiac surgery specialists based on the above-mentioned criteria.

Adverse events selected for follow-up included infections (driveline, pump pocket), pump thrombosis, bleeding, reoperation, ischemic and hemorrhagic stroke, and multiorgan failure. They were evaluated and recorded at the following time points: at 30 days after surgery, 3, 6, and 12 months, and at each subsequent yearly scheduled and unscheduled follow-up visit.

Mortality was defined as early, if occurring up to 30 days from implantation, or as late, if occurring after 30 days. The cause of death was specifically defined by a multidisciplinary consensus. In the situation where many severity comorbidities existed, one (main) cause of death was defined, and associated conditions that may affect mortality were categorized. In cases of fatal outcome, patient loss to follow-up, or heart transplant, the entire records up to the last follow-up visit were processed.

The complete statistical analysis was done with the statistical software package IBM SPSS, version 26.0. Attribute variables were presented as the frequency of certain categories, while the statistical significance of differences was evaluated with the Chi-square test or Fisher’s exact test where the frequency in a category was small. Numerical variables were presented as mean values with standard deviation, minimum-maximum, or median values with an interquartile range (25–75th percentile). The statistical significance of differences was tested with the Mann–Whitney test or independent samples *t*-test according to the distribution of data. The unadjusted patient survival was calculated using Kaplan–Meier plots (mean with 95% confidence interval) and *p*-values derived from the univariate log-rank (Mantel–Cox) test. The normality of the data was assessed using the Kolmogorov–Smirnov test. The association between potential risk factors and the fatal outcome was evaluated using a multivariable Cox proportional hazard regression analysis, showing the strength of association by hazard ratio (HR) with 95% confidence intervals (CIs). All the analyses were estimated at a *p* < 0.05 level of statistical significance.

## 3. Results

The study included a total of 92 patients with a mean age of 54.57 years (standard deviation 12.16). There were a total of 84 men and eight women, and no significant difference in age was found between the genders (men: 54.53 ± 12.16; women: 56.00 ± 12.85; independent samples *t*-test, *p* = 0.729).

Of the total number of patients, 40 (43.5%) died. The median overall survival of patients after LVAD implantation was 22 months (IQR 8.5–47.5 months). In the non-survivors’ arm, the time from LVAD implantation to fatal outcome was nine months (1–18 months), while in the survivors’ arm, the median time to the end of follow-up was 38.5 months (18.25–66.5 months). In the entire study population, survival rates were 88.04% at one month, 80.43% at six months, 70.65% at one year, and 61.96% at two years.

There was a significant difference in several variables between patients who died after LVAD implantation and those who survived to the end of the follow-up (Table 1). Among non-survivors, the cardiac index (CI) and cardiac output (CO) were significantly lower, while the pulmonary artery wedge pressure (PAWP) was significantly higher than in survivors. Furthermore, the non-survivors showed significantly higher creatinine and urea levels and a significantly lower glomerular filtration rate (eGFR). Lactate dehydrogenase (LDH) levels were also significantly higher than in non-survivors. Renal insufficiency before surgery was significantly more common in the non-survivors than survivors, and so was dialysis-dependent renal insufficiency.

A significant difference in the RHF complication rate after LVAD implantation between the two analyzed arms was found only for the development of early RHF (Table 1), which developed significantly more frequently in non-survivor patients compared to survivor patients (35.9% vs. 3.9%).

**Figure 1:**
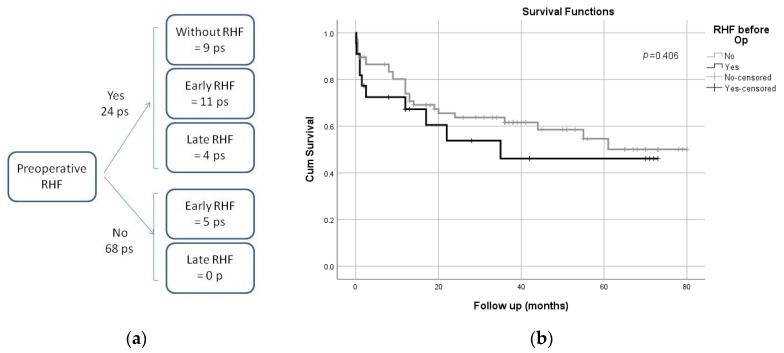
(**a**) Distribution of patients by RHF; preoperative, early, and late form; (**b**) survival analysis against pre-implantation RHF.

The preoperative RHF was present in 24 patients, 12 of whom died and 12 survived LVAD implantation (Figure 1a). Only one survivor developed an early RHF (ERHF) and two late RHFs (LRHF). However, as many as 10 non-survivors developed an early form of RHF, and two developed a late form.

The comparison of patient survival based on RHF shows that preoperative RHF did not affect patient survival (Figure 1b). The average survival was 39.90 months (95% CI: 25.26–54.54) and 50.28 (41.76–58.80) months in non-survivors and survivors, respectively. No significant difference in the survival rate was found between these two arms (log-rank (Mantel–Cox) test, *p* = 0.406). In the group of 24 subjects with preoperative RHF, 50% fatal outcomes were observed, while in the group without preoperative RHF, 28 (or 41.2%) were with fatal outcomes.

According to the cause of death, significant differences were found between the groups with and without postoperative development of RHF (Chi-square test; *p* = 0.001). In patients with post-operative RHF, the most common cause of death was RHF (7 or 43.8%), while other causes of death were multiorgan failure (MOF) (4 patients), sepsis or bleeding (both 2 patients), and CVI (one patient). In patients without RHF after LVAD implantation, the most common cause of death was CVI (10 or 41.7%), while other causes of death were sepsis (3 patients), thrombosis and bleeding (both 2 patients), MOF (one patient), and other causes (6 patients).

**Figure 2:**
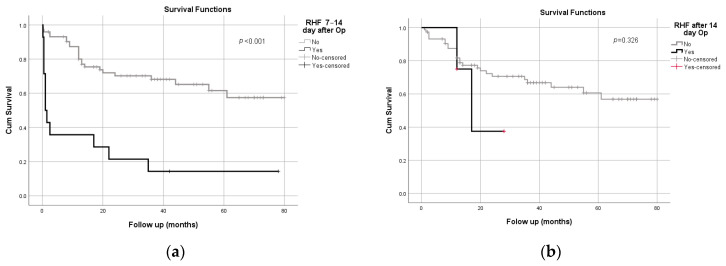
(**a**) Survival analysis against early RHF (7–14 days after LVAD implantation); (**b**) survival analysis against late RHF (>14 days after LVAD implantation).

However, the development of the early form of RHF significantly shortened patient survival (Figure 2a,b). In patients who developed early RHF, the survival time was significantly shorter than in patients who survived to the end of the follow-up period (non-survivors: 17.05 [2.97–31.13]; survivors: 55.66 [47.84]; log-rank [Mantel–Cox] test, *p* < 0.001), i.e., out of 16 patients with early RHF, 14 died, as well as 24 patients out of 73 who did not develop early RHF. On the other hand, patients who developed late RHF when compared to those who did not develop late RHF did not show a statistically significant difference in survival (non-survivors: 19.87 [12.63–27.12]; survivors: 55.53 [47.87–63.19]; log-rank [Mantel–Cox] test, *p* = 0.326), i.e., out of 4 patients with late RHF, 2 died, as well as 25 patients out of 74 who did not develop late RHF.

Of all the observed variables, the following patient features shown in Table 2 were identified as predictors of mortality. The most significant predictors were INTERMACS class, NYHA class, and the development of early RHF (HR: 0.018, 11.100, and 10.681, respectively).

The most significant predictors of early RHF development were BNP, pre-surgery RHF, FAC < 20%, prior renal insufficiency, and total duration of ICU stay (HR: 1.002, 0.901, 0.858, 23.554, and 1.005), all according to adjusted HR (Table 3).

No significant predictor of late RHF development in the adjusted HR proved to be significant, although there were several significant predictors in the unadjusted Cox proportional hazard regression analysis (dialysis-dependent renal insufficiency, right atrial pressure, total duration of ICU stays, pump thrombosis, and serum albumin) (Table 4).

There was a significant difference in several variables between 20 patients who developed the post-operative RHF (16 patients with early RHF and 4 patients with LRHF) and those who did not have RHF after LVAD implantation (Table 5). Among patients with post-operative RHF, mPAP, PAWP, right atrial pressure, CVP/PCWP score, BNP, total bilirubin, LDH, and duration of ICU stay were significantly higher, while the albumin and RV FAC% were significantly lower than in patients without RHF after LVAD implantation. Additionally, the patients with post-operative RHF showed significantly lower INTERMACS profiles (70% with values one and two). In addition, they were subjected to significantly more frequent administration of IV inotropic agents compared to those who did not develop RHF postoperatively (80% vs. 31.9%). Patients with post-operative RHF had pre-LVAD RHF (75% vs. 12.5%), FAC < 20% (30% vs. 8.3%), renal insufficiency (60% vs. 16.7%), and dialysis-dependent renal insufficiency (25% vs. 5.6%) significantly more often compared to those who did not develop RHF postoperatively. 

There was a significant difference in variables within 24 patients with pre-operative RHF considering the development of post-operative RHF after LVAD implantation (9 patients without postoperative RHF and 15 with postoperative RHF) (Table 6). Among patients with post-operative RHF, the CVP/PCWP score was significantly higher and eGFR was significantly lower than in patients without RHF following LVAD implantation. Additionally, dilated cardiomyopathy has been shown to be the most common cause of postoperative RHF in this isolated group. Although there were significant differences in the clinical sense, no statistically significant difference was shown between the other variables.

## 4. Discussion

Our findings suggest that patients with preoperative RHF are at increased risk for developing post-implantation RHF. The occurrence of RHF in the immediate post-implantation period or later is a proven factor contributing to a higher mortality rate. According to our results, the optimization of renal function, management of arrhythmias, and optimization of pulmonary circulation conditions may be identified as procedures that can help prevent an early and late RHF.

Since preoperative RHF was not a significant predictor of mortality in the study, and a significant number of patients experienced withdrawal of RHF symptoms following the LVAD implantation, this study tried to identify factors that may indicate the potential reversibility of RHF. In a sample of 24 patients with preoperative RHF, after LVAD implantation, the patients without RHF had a significantly lower CVP/PCWP score compared to patients with RHF. The etiology of dilated cardiomyopathy has been shown to be a significant predictor in this group because the primary pathological process affects the whole myocardium. In contrast to this fact, in patients with ischemic cardiomyopathy, the right ventricle often has preserved functions. Additionally, eGFR was lower in the group of patients in whom the RHF did not subside. 

LRHF was not common in our study and out of the four patients with LRHF, two survived to the intersection of the study. All four patients had preoperative RHF. Given that in all four patients LRHF occurred during the first year after implantation and that three patients were on the BTT list (and did not have a heart transplant), the LRHF condition can be viewed as a pathological process that occurs independently of the initial hemodynamic changes after implantation. In our sample, end-stage renal failure and pump thrombosis were the strongest predictors of LRHF development. However, as there are many more patients with LVAD devices implanted for many years, LRHF will be increasingly in the clinical focus [11,12]. 

Our results are consistent with previous studies that showed a significant association between post-implantation RHF and renal dysfunction [13,14]. Furthermore, our results did not show any significant difference in the incidence of RHF depending on gender, age, and body mass index (BMI). They show that the BNP level can be a useful prognostic indicator of the development of early RHF, but further studies will evaluate this. A low INTERMACS class and NYHA class IV (unlike NYHA class IIIB) were significant predictors of the development of post-implantation RHF in our study. The decision on the time of LVAD implantation was shown to be extremely important for potential prevention of post-implantation RHF [15]. 

Patients who develop RHF in the postoperative period are at risk of developing MOF, particularly of the respiratory and renal systems. The degree of multiorgan dysfunction in the preimplantation period is a quality predictor of pronounced postoperative RHF, and the results of our study correlate with these conclusions [16]. Some publications emphasize the association between elevated CVP and the risk of RHF [17]. We consider that the isolated CVP level, which in our research was one of the criteria for defining RHF, has even stronger prognostic value within the CVP/PCWP score. Among echocardiography parameters, low RV FAC (<20%) was shown to have the most significant predictive value for the development of early RHF, but the significance of this isolated echocardiographic parameter must be taken with extreme caution due to the potential influence of high PVR, the presence of secondary TR, and dynamic changes in volume load [18].

Various risk factors and risk scores are widely available to the medical public, but the optimal method for predicting RHF is still in the phase of intensive research [19,20]. The CVP/PCWP score in our study confirmed the significance in relation to the threshold value of 0.63 when significant predictors of RHF are identified [21]. In the group of patients with preoperative RHF, the CVP/PCWP scores were significantly higher in patients in whom the RHF did not subside after LVAD implantation.

Furthermore, the method of diagnosis of RHF and the severity of the clinical picture are still the subject of debate due to the inconsistency of the evaluated and published parameters (echocardiographic, hemodynamic, clinical, and laboratory).

The effect of LVAD device activation may be considered to be a complex result of different effects on heart contractility, RV preload and afterload, and long-term altered overall circulatory conditions of continuous blood flow [22].

Fixation of the apex of the heart with the inflow cannula can alter the normal twisting contraction of the RV [23]. On the one hand, accelerated and facilitated LV emptying due to pump operation increases the inflow into the RV, and, due to such volume load, the septum shifts to the left, affecting the shape, size, and function of the right ventricle, as well as the subsequent filling of LV. On the other hand, the facilitated emptying reduces pulmonary congestion and right ventricular afterload [24,25].

From the above, it follows that a balance is required between treatment procedures related to the intensity of pump operation, inotropic support, maintenance of volume requirements, and treatment of possible pulmonary hypertension in the post-implantation period.

The development of the LVAD program has been intensified in several directions, aimed at reducing the incidence of adverse events [26]. 

The development of fully implantable LVAD devices is expected to significantly reduce the incidence of LVAD-specific infections [27,28]. According to early reports, new devices with levitating systems show a reduced rate of complications, such as stroke and hemorrhage [29,30]. Such programs and a subsequent reduction of the incidence of the above-mentioned adverse events may significantly increase the relative rate of post-implantation RHF as a proportion of the total number of complications. This requires intensive research of various strategies for the timely identification of patients at risk of post-implantation RHF, and preventive and therapeutic procedures.

Considering that arrhythmias are identified as an inclining factor for RHF throughout the implant surgery, we consider avoiding suturing coronary blood vessels near the apex of the heart during fixation of the pump ring to be a useful prevention strategy because we believe that maintaining the optimal coronary flow is vital for RV function. Our practice did not involve the concomitant coronary artery bypass graft (CABG) at the time of LVAD implantation, based on publications reporting no significant difference in the incidence of postoperative arrhythmias and RHF in patients with surgical revascularization at the time of LVAD implantation [31,32].

Tricuspid insufficiency categorized as 3+ or greater is a significant factor for the occurrence of postoperative RHF. On the other hand, tricuspid valve (TV) surgery was not identified as a predisposing factor for the development of RHF; however, due to the small sample, an adequate interpretation of the potential benefits of TV surgery is limited. The importance of management of tricuspid insufficiency must be evaluated in future research [33].

Many cardiac surgery centers practice an alternative method of LVAD implantation via lateral thoracotomy with preservation of the pericardium, which maintains the RV geometry, prevents distension, and avoids RV compression that is present with sternotomy. The mentioned approach was reported to show potential benefits for post-implantation functioning of the right ventricle [34].

Different right ventricular assist devices (RVADs) may be an effective treatment solution for refractory post-implantation RHF [35]. The efficacy of preoperative RVAD system implantation to prevent RHF is increasingly evaluated. It has been shown that the peripheral placement of new-generation devices that can perform RVAD function without resternotomy is a particularly suitable option with a reduced incidence of postoperative complications and better clinical outcomes [36].

The new-generation devices for short-term and intermediate mechanical circulatory support characterized by an advanced design and introduced by peripheral vascular approach may, in addition to survival benefits until implantation of a definitive LVAD system, be used to create the actual temporary hemodynamic conditions that clearly show the effect of LVAD activation on RV. This enables timely, optimal, and personalized treatment during the post-implantation period [37]. This strategy may be described as a direct assessment of the reversibility of RV damage but requires additional research that will evaluate the potential therapeutic benefit taking into account any potential complications due to implantation of a temporary device and possibly increased treatment costs.

Limitations: This was a single-site cohort study that included 92 treated patients. The results must be interpreted with caution in terms of drawing generalized clinical conclusions. Patients were implanted with three different devices, and no significant difference was found in the incidence of RHF among these particular groups, but this may be characterized as a limiting factor. This study did not evaluate any intraoperative parameters. No conclusions about temporary RVAD support can be made from this study because it was administered in one patient only. In addition to the above-listed advantages, the definition we used may provide a different RHF incidence rate compared to other publications and does not categorize the severity of the RHF clinical picture.

## 5. Conclusions

RHF following LVAD implantation is an unwanted complication with a negative impact on treatment outcomes. Preoperative RHF has not been shown to be a significant mortality factor. The results of this study indicate that there are a significant number of patients in whom preoperative RHF improves after LVAD implantation. Assessing whether RV is irreversibly damaged to the point where it cannot respond to activation by an LVAD device requirements is a major clinical challenge. The etiology of cardiomyopathy is a significant predictive factor, and the CVP/PCWP score has confirmed prognostic value in this aspect (in this group of patients with reversible RHF after LVAD implantation) as well. The increased risk of fatal outcome in patients with ERHF and LRHF after LVAD implantation results in a need to administer available preventive and therapeutic methods. Preoperative renal dysfunction, high BNP level, and FAC < 20% are the most significant predictors of ERHF. We did not identify LRHF as a common event, which implies that after the initial successful adaptation of all organ systems to the operating conditions of the LVAD device, RV will function adequately for a longer period. Our results may be part of a larger clinical mosaic of identifying the risk factors for RHF following LVAD implantation and thus requires further research.

## Figures and Tables

**Table 1:** Patient socio-demographic and clinical features—baseline data.

	All Patients (n = 92)	Survivors (n = 52)	Non-Survivors (n = 40)	*p* Value
Age at the time of LVAD implantation	54.57 ± 12.16	53.13 ± 12.86	56.43 ± 11.06	0.200 *
Gender: female/male	84 (91.3)/8 (8.7)	48 (92.3)/4 (7.7)	36(90.0)/4(10.0)	0.987 **
LVAD/heart transplant (HTx) after LVAD	86 (93.5)/6 (6.5)	48 (92.3)/4 (7.7)	38(95.0)/2(5.0)	0.926 **
Heart Mate II/Heart Mate III/HeartWare	33 (35.9)/26 (28.3)/33 (35.9)	18 (34.6)/18 (34.6)/16 (30.8)	15 (37.5)/8 (20.0)/17 (42.5)	0.269 **
LVAD + tricuspid valve surgery	87 (94.6)/5 (5.4)	2 (3.8)	3 (7.5)	0.762 **
LVAD + aortic valve surgery	87 (94.6)/5 (5.4)	5 (9.6)	0	0.120 **
Implantation urgency	84 (91.3)/8 (8.7)	4 (7.7)	4 (10.0)	0.987 **
REDO before LVAD	82 (89.1)/10 (10.9)	4 (7.7)	6 (15.0)	0.436 **
Heart transplant after LVAD	6 (6.5)	4 (7.7)	2 (5.0)	0.926 **
Time from LVAD to HTx	13–36	13–36	13–23	0.800 ^#^
BTT on the list for HTx	64 (69.6)	36 (69.2)	28 (70.0)	1.000 **
Etiology: ischemic CMP/dilated CMP/viral myocarditis/postpartum CMP/noncompaction CMP	45 (48.9)/36 (39.1)/9 (9.8)/1 (1.1)/1 (1.1)	25 (48.1)/18 (34.6)/7 (13.5)/1 (1.9)/1 (1.9)	20 (50.0)/18 (45.0)/2 (5.0)/0/0	0.429 **
NYHA III/IV	7 (7.6)/85 (92.4)	4 (7.7)/48 (92.3)	3 (7.5)/37 (92.5)	1.000 **
INTERMACS profile: 1–2/3–4/5	27 (29.3)/55 (59.8)/10 (10.9)	13 (25.0)/34 (65.4)/5 (9.6)	14 (35.0)/21 (52.5)/5 (12.5)	0.635 **
Body mass index (BMI)	25.30 ± 3.87	25.16 ± 2.78	25.46 ± 4.94	0.803 *
LV EF (%)	16.70 ± 5.09	16.54 ± 4.91	16.90 ± 5.38	0.738 *
LV EDD (cm)	7.75 ± 1.27	7.80 ± 1.03	7.68 ± 1.55	0.665 *
LV ESD (cm)	6.80 ± 1.07	6.96 ± 1.05	6.58 ± 1.08	0.094 *
CI (L/min/m^2^)	2.14 ± 0.48	2.22 ± 0.52	2.02 ± 0.40	0.045 *
CO (L/min)	4.09 ± 0.99	4.23 ± 1.09	3.84 ± 0.79	0.031 *
mPAP (mmHg)	31.60 ± 8.90	30.25 ± 8.36	33.35 ± 9.36	0.098 *
PAWP (mmHg)	21.59 ± 6.95	20.17 ± 6.92	23.42 ± 6.62	0.025 *
PVR (WU)	2.51 ± 1.66	2.29 ± 1.59	2.78 ± 1.74	0.161 *
Right atrial pressure (mmHg)	11.46 ± 5.42	9.50 (7.00–16.00)	11.50 (8.00–16.75)	0.224 ^#^
TPG (mmHg)	10.36 ± 5.60	11.00 (6.00–13.00)	10.50 (6.25–13.00)	0.997 ^#^
CVP/PCWP score	0.53 ± 0.27	0.56 ± 0.32	0.50 ± 0.19	0.319 *
CVP/PCWP score: <0.63/>0.63	64 (69.6)/28 (30.4)	34 (65.4)/18 (34.6)	30 (75.0)/10 (25.0)	0.444 **
BNP (pg/mL)	1196.50 (573.25–2371.25)	1238.50 (577.00–2203.25)	970.50 (511.25–2486.00)	0.984 ^#^
Sodium (mmol/L)	135.46 ± 4.08	135.50 ± 3.92	135.40 ± 4.32	0.908 *
Albumin (g/L)	38.41 ± 6.23	38.50 ± 6.94	38.30 ± 5.26	0.880 *
Creatinine (µmol/L)	116.61 ± 60.80	102.92 ± 34.23	134.40 ± 80.73	0.013 *
Urea (mmol/L)	10.07 ± 5.88	8.49 ± 3.58	12.14 ± 7.51	0.003 *
eGFR (mL/min /1.73 m^2^)	52.17 ± 11.79	54.37 ± 8.70	49.33 ± 14.51	0.041 *
Alanine aminotransferase (ALT) (U/L)	24.00 (18.25–36.75)	24.50 (19.25–37.00)	24.00 (15.00–35.75)	0.801 ^#^
Aspartate aminotransferase (AST) (U/L)	25.00 (20.00–35.00)	24.50 (19.00–35.00)	25.50 (20.25–34.75)	0.619 ^#^
Total bilirubin (µmol/L)	21.60 (14.00–42.00)	21.60 (14.00–39.00)	21.75 (14.22–50.75)	0.504 ^#^
Lactate dehydrogenase (LDH)	449.12 ± 122.36	427.23 ± 99.23	477.58 ± 143.49	0.050 *
IV inotropic agents	39 (42.4)	21 (40.4)	18 (45.0)	0.817 **
ICD/CRT implantation before LVAD	49 (53.3)	24 (46.2)	25 (62.5)	0.178 **
Absolute arrhythmia	46 (50.0)	21 (40.4)	25 (62.5)	0.058 **
Diabetes mellitus	21 (22.8)	12 (23.1)	9 (22.5)	1.000 **
Hypertension	36 (39.1)	23 (44.2)	13 (32.5)	0.354 **
Bleeding after LVAD	36 (39.1)	23 (44.2)	13 (32.5)	0.354 **
Pre-LVAD RHF	24 (26.1)	12 (23.1)	12 (30.0)	0.610 **
Right ventricular systolic pressure (RVSP) (mmHg)	48.30 ± 16.44	47.10 ± 17.92	49.88 ± 14.27	0.425 *
FAC < 20%	12 (13.0)	7 (13.5)	5 (12.5)	1.000 **
RV FAC%	28.77 ± 10.01	29.25 ± 10.69	28.15 ± 9.14	0.604 *
RV cm	3.00 (2.50–3.60)	3.00 (2.42–3.57)	3.00 (2.72–3.70)	0.203 ^#^
Tricuspid regurgitation (TR): 0/1+/2+/3+ or greater	1 (1.1)/30 (32.6)/34 (37.0)/27 (29.3)	1 (1.9)/15 (28.8)/23 (44.2)/13 (25.0)	0/15 (37.5)/11 (27.5)/14 (35.0)	0.287 **
RV TAPSE (mm)	17.46 ± 4.50	17.88 ± 5.22	16.90 ± 3.33	0.301 *
RV Sm of T annulus (cm/s)	10.79 ± 2.67	10.98 ± 2.71	10.54 ± 2.64	0.434 *
Renal insufficiency	24 (26.1)	9 (17.3)	15 (37.5)	0.029 **
Dialysis-dependent renal insufficiency	9 (9.8)	1 (1.9)	8 (20.0)	0.012 **
Driveline infection	3 (3.3)	1 (1.9)	2 (5.00)	0.817 **
Bleeding during LVAD	36 (39.1)	21 (40.4)	15 (37.5)	0.948 **
Reoperation due to bleeding during LVAD	20 (21.7)	12 (23.1)	8 (20.0)	0.921 **
CVI after LVAD	15 (16.3)	5 (9.6)	10 (25.6)	0.080 **
Pump thrombosis	10 (10.9)	3 (5.8)	7 (17.5)	0.146 **
Total duration of hospital stay after LVAD	25.50 (20.00–31.75)	27.50 (21.25–32.00)	23.50 (17.00–30.00)	0.059 ^#^
Duration of ICU stay	9.00 (7.00–16.00)	9.00 (7.00–15.00)	9.00 (6.25–17.00)	0.613 ^#^
Early RHF	16 (17.4)	2 (3.9)	14 (35.9)	<0.001 **
Late RHF	4 (4.3)	2 (3.9)	2 (7.4)	0.901 **

The data are presented as absolute numbers (%), mean ± standard deviation, value range (minimum-maximum value), or median with IQR (interquartile range: 25–75 percentile); * Independent samples *t*-test; ** Chi-square test; ^#^ Mann-Whitney test.

**Table 2:** Mortality predictors.

	Unadjusted HR (95% CI)	*p* Value	Adjusted HR (95% CI)	*p* Value
Implantation urgency	4.188 (1.426–12.298)	0.009	3.979 (1.148–13.797)	0.029
LVAD + aortic valve surgery	3.016 (1.161–7.835)	0.023	-	-
INTERMACS class	3.652 (1.212–11.002)	0.021	11.100 (2.653–46.437)	0.001
NYHA	0.041 (0.010–0.165)	<0.001	0.018 (0.002–0.138)	<0.001
PAWP (mmHg)	0.956 (0.918–0.995)	0.029	-	-
TPG (mmHg)	1.060 (1.012–1.111)	0.015	-	-
Bleeding after LVAD	0.500 (0.259–0.962)	0.038	0.372 (0.175–0.791)	0.010
Pre-LVAD renal insufficiency	2.912 (1.317–6.441)	0.008	-	-
Early RHF	3.658 (1.815–16.407)	0.050	10.681 (1.872–60.949)	0.008

Cox proportional hazard regression analysis; HR: Hazard ratio.

**Table 3:** Predictors of early RHF development.

	Unadjusted HR (95% CI)	*p* Value	Adjusted HR (95% CI)	*p* Value
INTERMACS profile	0.229 (0.101–0.519)	<0.001	-	-
mPAP	1.058 (1.002–1.116)	0.042	-	-
PAWP	1.089 (1.006–1.179)	0.036	-	-
Right atrial pressure	1.181 (1.075–1.298)	0.001	-	-
BNP	1.001 (1.000–1.001)	<0.001	1.002 (1.000–1.001)	0.028
Creatinine	1.006 (1.002–1.011)	0.008	-	-
Urea	1.072 (1.001–1.148)	0.048	-	-
eGFR	0.952 (0.919–0.985)	0.005	-	-
ALT	1.009 (1.004–1.014)	<0.001	-	-
AST	1.015 (1.005–1.025)	0.002	-	--
Bilirubin, total	1.027 (1.011–1.044)	0.001	-	-
LDH	1.006 (1.003–1.010)	<0.001	-	-
IV inotropic agents	18.987 (2.464–146.315)	0.005	-	-
Absolute arrhythmia	8.315 (1.819–38.004)	0.006	-	-
Pre-surgery RHF	7.568 (2.354–24.915)	0.001	0.901 (0.101–0.998)	0.050
FAC < 20%	0.901 (0.842–0.998)	0.032	0.858 (0.653–0.999)	0.043
RV Sitolic motion of tricuspid annulus	0.787 (0.637–0.973)	0.027	-	-
Prior renal insufficiency	26.610 (5.545–127.685)	<0.001	23.554 (5.005–201.359)	<0.001
	1.098 (1.053–1.114)	<0.001	1.005 (1.001–1.352)	0.049

Cox proportional hazard regression analysis; HR: Hazard ratio.

**Table 4:** Predictors of late RHF development.

	Unadjusted HR (95% CI)	*p* Value	Adjusted HR (95% CI)	*p* Value
Right atrial pressure	1.251 (1.050–1.489)	0.012	-	-
Serum albumin	0.888 (0.823–0.958)	0.002	-	-
Dialysis-dependent renal insufficiency	22.034 (2.980–162.906)	0.002	-	-
Pump thrombosis	13.326 (1.833–96.894)	0.011	-	-
Total duration of ICU stay	1.136 (1.039–1.241)	0.005	-	-

Cox proportional hazard regression analysis; HR: Hazard ratio.

**Table 5:** Comparison of data of patients between who developed post-operative RHF with those who did not have RHF after LVAD implantation.

	All Patients (n = 92)	Without RHF (n = 72)	With RHF (n = 20)	*p* Value
Age at the time of LVAD implantation	54.57 ± 12.16	55.28 ± 11.81	52.00 ± 13.33	0.289 *
Gender: female/male	84 (91.3)/8 (8.7)	66 (91.7)/6 (8.3)	18(90.0)/2(10.0)	1.000 **
LVAD/heart transplant (HTx) after LVAD	86 (93.5)/6 (6.5)	66 (91.7)/6 (8.3)	20(100.0)/0(0.0)	0.410 **
Heart Mate II/Heart Mate III/HeartWare	33 (35.9)/26 (28.3)/33 (35.9)	26 (36.1)/19 (26.4)/27 (37.5)	7 (37.0)/7 (35.0)/6 (30.0)	0.722 **
LVAD + tricuspid valve surgery	87 (94.6)/5 (5.4)	2 (2.8)	3 (15.0)	0.115 **
LVAD + aortic valve surgery	87 (94.6)/5 (5.4)	5 (6.9)	0	0.513 **
Implantation urgency	84 (91.3)/8 (8.7)	4 (5.6)	4 (20.0)	0.114 **
REDO before LVAD	82 (89.1)/10 (10.9)	8 (11.1)	2 (10.0)	1.000 **
Heart transplant after LVAD	6 (6.5)	6 (8.3)	0	0.410 **
Time from LVAD to HTx	13–36	13–36	/	
BTT on the list for HTx	64 (69.6)	49 (68.1)	15 (75.0)	0.747 **
Etiology: ischemic CMP/dilated CMP/viral myocarditis/postpartum CMP/noncompaction CMP	45 (48.9)/36 (39.1)/9 (9.8)/1 (1.1)/1 (1.1)	39 (54.2)/23 (31.9)/8 (11.1)/1 (1.4)/1 (1.4)	6 (30.0)/13 (65.0)/1 (5.0)/0/0	0.106 **
NYHA III/IV	7 (7.6)/85 (92.4)	7 (9.7)/65 (90.3)	0/20 (100.0)	0.330 **
INTERMACS profile: 1–2/3–4/5	27 (29.3)/55 (59.8)/10 (10.9)	13 (18.1)/49 (68.0)/10 (13.9)	14 (70.0)/6 (30.0)/0	<0.001 **
Body mass index (BMI)	25.30 ± 3.87	25.61 ± 3.52	23.90 ± 5.21	0.264 *
LV EF (%)	16.70 ± 5.09	16.82 ± 4.79	16.25 ± 6.18	0.661 *
LV EDD (cm)	7.75 ± 1.27	7.72 ± 1.01	7.86 ± 1.98	0.668 *
LV ESD (cm)	6.80 ± 1.07	6.86 ± 1.06	6.58 ± 1.11	0.320 *
CI (L/min/m^2^)	2.14 ± 0.48	2.14 ± 0.49	2.12 ± 0.46	0.865 *
CO (L/min)	4.09 ± 0.99	4.12 ± 0.98	3.98 ± 1.02	0.583 *
mPAP (mmHg)	31.60 ± 8.90	30.39 ± 8.02	35.95 ± 10.64	0.013 *
PAWP (mmHg)	21.59 ± 6.95	20.32 ± 6.54	26.15 ± 6.60	0.001 *
PVR (WU)	2.51 ± 1.66	2.43 ± 1.77	2.77 ± 1.19	0.420 *
Right atrial pressure (mmHg)	11.46 ± 5.42	9.00 (7.00–12.00)	18.00 (16.00–19.00)	<0.001 ^#^
TPG (mmHg)	10.36 ± 5.60	11.00 (6.00–13.00)	11.00 (5.50–13.00)	0.955 ^#^
CVP/PCWP score	0.53 ± 0.27	0.50 ± 0.28	0.64 ± 0.19	0.044 *
CVP/PCWP score: <0.63/>0.63	64 (69.6)/28 (30.4)	55 (76.4)/17 (23.6)	9 (45.0)/11 (55.0)	0.015 **
BNP (pg/mL)	1196.50 (573.25–2371.25)	968.00 (409.00–1617.75)	2648.50 (1348.75–3608.50)	<0.001 ^#^
Sodium (mmol/L)	135.46 ± 4.08	135.47 ± 3.53	135.40 ± 5.75	0.945 *
Albumin (g/L)	38.41 ± 6.23	39.14 ± 4.97	35.80 ± 9.12	0.033 *
Creatinine (µmol/L)	116.61 ± 60.80	111.22 ± 56.98	136.00 ± 71.18	0.107 *
Urea (mmol/L)	10.07 ± 5.88	9.79 ± 5.48	11.09 ± 7.26	0.395 *
eGFR (mL/min /1.73 m^2^)	52.17 ± 11.79	53.42 ± 10.56	47.70 ± 14.89	0.055 *
Alanine aminotransferase (ALT) (U/L)	24.00 (18.25–36.75)	24.00 (18.00–34.75)	31.50 (20.00–60.50)	0.193 ^#^
Aspartate aminotransferase (AST) (U/L)	25.00 (20.00–35.00)	25.00 (19.25–34.00)	27.00 (21.25–52.50)	0.274 ^#^
Total bilirubin (µmol /L)	21.60 (14.00–42.00)	19.10 (13.10–31.30)	48.40 (23.47–62.82)	<0.001 ^#^
Lactate dehydrogenase (LDH)	449.12 ± 122.36	427.76 ± 110.15	526.00 ± 135.72	0.001 *
IV inotropic agents	39 (42.4)	23 (31.9)	16 (80.0)	<0.001 **
ICD/CRT implantation before LVAD	49 (53.3)	37 (51.4)	12 (60.0)	0.668 **
Absolute arrhythmia	46 (50.0)	32 (44.4)	14 (70.0)	0.077 **
Diabetes mellitus	21 (22.8)	18 (25.0)	3 (15.0)	0.521 **
Hypertension	36 (39.1)	31 (43.1)	5 (25.0)	0.228 **
Bleeding after LVAD	36 (39.1)	27 (37.5)	9 (45.0)	0.727 **
Pre-LVAD RHF	24 (26.1)	9 (12.5)	15 (75.0)	<0.001 **
Right ventricular systolic pressure (RVSP) (mmHg)	48.30 ± 16.44	47.82 ± 17.34	50.05 ± 12.92	0.594 *
FAC < 20%	12 (13.0)	6 (8.3)	6 (30.0)	0.030 **
RV FAC%	28.77 ± 10.01	29.97 ± 9.72	24.45 ± 10.08	0.028 *
RV cm	3.00 (2.50–3.60)	3.30 (2.80–3.50)	3.50 (3.00–3.87)	0.114 ^#^
Tricuspid regurgitation (TR): no/1+/2+/3+ or greater	1 (1.1)/30 (32.6)/34 (37.0)/27 (29.3)	1 (1.4)/29 (40.3)/25 (34.7)/17 (23.6)	0/1 (5.0)/9 (45.0)/10 (50.0)	0.015 **
RV TAPSE (mm)	17.46 ± 4.50	17.74 ± 4.29	16.45 ± 5.18	0.261 *
RV Sm of T annulus (cm/s)	10.79 ± 2.67	11.04 ± 2.34	9.88 ± 3.55	0.085 *
Renal insufficiency	24 (26.1)	12 (16.7)	12 (60.0)	<0.001 **
Dialysis-dependent renal insufficiency	9 (9.8)	4 (5.6)	5 (25.0)	0.032 **
Driveline infection	3 (3.3)	3 (4.2)	0	0.829 **
Bleeding during LVAD	36 (39.1)	28 (38.9)	8 (40.0)	1.000 **
Reoperation due to bleeding during LVAD	20 (21.7)	15 (20.8)	5 (25.0)	0.926 **
CVI after LVAD	15 (16.3)	13 (18.1)	2 (10.5)	0.660 **
Pump thrombosis	10 (10.9)	7 (9.7)	3 (15.0)	0.791 **
Total duration of hospital stay after LVAD	25.50 (20.00–31.75)	26.00 (21.00–31.00)	22.00 (15.50–34.75)	0.437 ^#^
Duration of ICU stay	9.00 (7.00–16.00)	8.00 (6.00–13.75)	18.50 (10.00–26.75)	<0.001 ^#^
Early RHF	16 (17.4)	0	16 (84.2)	<0.001 **
Late RHF	4 (4.3)	0	4 (36.4)	<0.001 **

The data are presented as absolute numbers (%), mean ± standard deviation, value range (minimum-maximum value) or median with IQR (interquartile range: 25–75 percentile); * Independent samples *t*-test; ** Chi-square test; ^#^ Mann–Whitney test.

**Table 6:** Comparison of data in the 24 patients with pre-operative RHF by the development of postoperative RHF after LVAD implantation.

	All Patients (n = 24)	Without Postoperative RHF (n = 9)	With Postoperative RHF (n = 15)	*p* Value
Age at the time of LVAD implantation	47.83 ± 13.97	41.44 ± 15.21	51.67 ± 12.10	0.082 *
Gender: female/male	23 (95.8)/1 (4.2)	9 (100.0)/0	14 (93.3)/1 (6.7)	1.000 **
LVAD/heart transplant (HTx) after LVAD	23 (95.8)/1 (4.2)	8 (88.9)/1(11.1)	15 (100.0)/0	0.792 **
Heart Mate II/Heart Mate III/HeartWare	10 (41.7)/7 (29.2)/7 (29.2)	4 (44.4)/3 (33.3)/2 (22.2)	6 (40.0)/4 (26.7)/5 (33.3)	0.836 **
LVAD + tricuspid valve surgery	4 (16.7)	1 (11.1)	3 (75.0)	1.000 **
LVAD + aortic valve surgery	0	0	0	
Implantation urgency	5 (20.8)	1 (11.1)	4 (26.7)	0.697 **
REDO before LVAD	2 (8.3)	0	2 (13.3)	0.703 **
Heart transplant after LVAD	1 (4.2)	1 (11.1)	0	0.792 **
Time from LVAD to HTx	13	13	/	
BTT on the list for HTx	19 (79.2)	8 (88.9)	11 (73.3)	0.697 **
Etiology: ischemic CMP/dilated CMP/viral myocarditis	6 (25.0)/13 (54.2)/5 (20.8)	1 (11.1)/4 (44.4)/4 (44.4)	5 (33.3)/9 (60.0)/1 (6.7)	0.050 **
NYHA IV	24 (100.0)	9 (100.0)	15 (100.0)	
INTERMACS profile: 1–2/3–4	18 (75.0)/6 (25.0)	6 (66.7)/3 (33.3)	12 (80.0)/3 (20.0)	0.506 **
Body mass index (BMI)	24.54 ± 4.77	24.91 ± 4.10	24.28 ± 5.51	0.834 *
LV EF (%)	14.71 ± 4.75	13.11 ± 3.65	15.67 ± 5.18	0.209 *
LV EDD (cm)	7.66 ± 1.13	8.14 ± 0.96	7.37 ± 1.16	0.104 *
LV ESD (cm)	6.97 ± 1.17	7.47 ± 0.95	6.65 ± 1.22	0.104 *
CI (L/min/m^2^)	2.23 ± 0.69	2.36 ± 0.98	2.15 ± 0.48	0.502 *
CO (L/min)	4.24 ± 1.23	4.49 ± 1.57	4.09 ± 1.02	0.459 *
mPAP (mmHg)	33.13 ± 9.88	29.00 ± 6.61	35.60 ± 10.86	0.115 *
PAWP (mmHg)	24.37 ± 7.31	20.78 ± 7.56	26.53 ± 6.47	0.060 *
PVR (WU)	2.23 ± 1.06	1.78 ± 1.16	2.50 ± 0.94	0.113 *
Right atrial pressure (mmHg)	18.00 (16.00–19.75)	16.00 (16.00–19.00)	19.00 (17.00–20.00)	0.155 ^#^
TPG (mmHg)	8.50 (4.25–12.00)	6.00 (3.00–12.50)	9.00 (5.00–12.00)	0.446 ^#^
CVP/PCWP score	0.80 ± 0.30	0.98 ± 0.41	0.69 ± 0.14	0.023 *
CVP/PCWP score: <0.63/>0.63	7 (29.2)/17 (70.8)	2 (22.2)/7 (77.8)	5 (33.3)/10 (66.7)	0.908 **
BNP (pg/mL)	2611.00 (1351.00–4003.00)	2699.00 (1205.00–3959.00)	2523.00 (1663.00–4120.00)	0.815 ^#^
Sodium (mmol/L)	135.29 ± 5.30	135.78 ± 3.96	135.00 ± 6.08	0.736 *
Albumin (g/L)	36.17 ± 8.99	38.78 ± 6.38	34.60 ± 10.13	0.280 *
Creatinine (µmol/L)	121.33 ± 61.87	97.56 ± 35.93	135.60 ± 70.49	0.149 *
Urea (mmol/L)	10.15 ± 6.71	8.34 ± 4.73	11.23 ± 7.60	0.318 *
eGFR (mL/min /1.73 m^2^)	51.04 ± 12.41	56.33 ± 5.66	47.87 ± 14.36	0.049 *
Alanine aminotransferase (ALT) (U/L)	31.00 (20.75–60.50)	38.00 (24.50–72.50)	24.00 (15.00–66.00)	0.318 ^#^
Aspartate aminotransferase (AST) (U/L)	26.00 (22.00–57.50)	44.00 (23.50–65.00)	24.00 (21.00–53.00)	0.379 ^#^
Total bilirubin (µmol /L)	49.90 (31.72–69.97)	36.90 (27.65–71.15)	52.00 (33.00–68.10)	0.682 ^#^
Lactate dehydrogenase (LDH)	488.33 ± 137.08	461.00 ± 122.18	504.73 ± 146.88	0.462 *
IV inotropic agents	22 (91.7)	9 (100.0)	13 (86.7)	0.703 **
ICD/CRT implantation before LVAD	13 (54.2)	5 (55.6)	8 (53.3)	1.000 **
Absolute arrhythmia	13 (54.2)	3 (33.3)	10 (66.7)	0.245 **
Diabetes mellitus	3 (12.5)	1 (11.1)	2 (13.3)	1.000 **
Hypertension	5 (20.8)	1 (11.1)	4 (26.7)	0.697 **
Bleeding after LVAD	12 (50.0)	4 (44.4)	8 (53.3)	1.000 **
Pre-LVAD RHF	24 (100.0)	9 (100.0)	15 (100.0)	
Right ventricular systolic pressure (RVSP) (mmHg)	48.04 ± 14.64	44.89 ± 15.28	49.93 ± 14.43	0.426 *
FAC < 20%	10 (41.7)	4 (44.4)	6 (40.0)	1.000 **
RV FAC%	22.92 ± 10.06	22.22 ± 8.00	23.33 ± 11.37	0.800 *
RV cm	3.80 (3.42–4.60)	4.20 (3.45–4.80)	3.70 (3.10–4.20)	0.318 ^#^
Tricuspid regurgitation (TR): 1+/2+/3+ or greater	1 (4.2)/9 (37.5)/14 (58.3)	1 (11.1)/3 (33.3)/5 (55.6)	0/6 (40.0)/9 (60.0)	0.416 **
RV TAPSE (mm)	15.96 ± 4.54	15.67 ± 1.73	16.13 ± 5.67	0.814 *
RV Sm of T annulus (cm/s)	9.75 ± 3.11	10.53 ± 2.06	9.29 ± 3.59	0.354 *
Renal insufficiency	11 (45.8)	2 (22.2)	9 (60.0)	0.169 **
Dialysis-dependent renal insufficiency	5 (20.8)	0	5 (33.3)	0.153 **
Driveline infection	0	0	0	
Bleeding during LVAD	13 (54.2)	5 (55.6)	8 (53.3)	1.000 **
Reoperation due to bleeding during LVAD	8 (33.3)	3 (33.3)	5 (33.3)	1.000 **
CVI after LVAD	2 (8.3)	0	2 (13.3)	0.703 **
Pump thrombosis	2 (8.3)	0	2 (13.3)	0.703 **
Total duration of hospital stay after LVAD	25.00 (19.00–30.75)	25.00 (19.00–27.50)	25.00 (17.00–35.00)	0.558 ^#^
Duration of ICU stay	15.00 (9.00–24.50)	15.00 (7.50–16.50)	17.00 (9.00–30.00)	0.215 ^#^
Early RHF	11 (45.8)	0	11 (78.6)	0.001 **
Late RHF	4 (16.7)	0	4 (40.0)	0.116 **

The data are presented as absolute numbers (%), mean ± standard deviation, value range (minimum-maximum value), or median with IQR (interquartile range: 25–75 percentile); * Independent samples *t*-test; ** Chi-square test; ^#^ Mann–Whitney test.

## Data Availability

The original contributions generated for the study are included in the article, further inquiries can be directed to the corresponding author/s.
